# Supplementary material for: Understanding networks in rural Cambodian farming communities and how they influence antibiotic use: A mixed methods study
Source: PLOS Glob Public Health. 2023 Mar 8;3(3):e0001569. doi: 10.1371/journal.pgph.0001569 (PMC10021636; doi:10.1371/journal.pgph.0001569)
Supplement: S1 Table — (PDF) [file pgph.0001569.s001.pdf]

S1 TABLE: PARTICIPANT ANTIBIOTIC SCORES

Each participant was assigned an antibiotic score, consisting of ten variables that elicited information about their antibiotic attitudes and practices. Each favourable response was scored 1 point, while a non-favourable response was scored 0 points. Participants' median antibiotic scores were 7.01 (range: 0 – 10).

**Favourable response = 1 point;**<br>**Non-favourable response = 0 points**	
1	I have heard of antibiotics/any of the brand names
2	I have gotten a prescription before getting antibiotics (for themselves)
3	I have gotten a prescription before getting antibiotics (for their animals)
4	I have kept a supply of antibiotics at home whether or not I'm sick
5	I have kept leftover antibiotics for future use
6	I have shared antibiotics with family and friends with the **same** symptoms
7	I have shared antibiotics with family and friends with **different** symptoms
8	I have shared antibiotics with my **animals**
9	I have used antibiotics to help my animals grow faster
10	I have used antibiotics to prevent illness in my animals
