# Supplementary material for: Understanding networks in rural Cambodian farming communities and how they influence antibiotic use: A mixed methods study
Source: PLOS Glob Public Health. 2023 Mar 8;3(3):e0001569. doi: 10.1371/journal.pgph.0001569 (PMC10021636; doi:10.1371/journal.pgph.0001569)
Supplement: S1 Appendix — (PDF) [file pgph.0001569.s002.pdf]

S1 APPENDIX: PHOTOGRAPHIC CENSUS DEMOGRAPHIC QUESTIONNAIRE

**Start baseline questionnaire**		
1.1	Name of data collector	
1.2	GPS of participant's home	
1.3	Time start	
1.4	Date	
1.5	Village	1) Village 1<br>2) Village 2
1.6	Household ID	Scan QR code
1.7	How many people in this household would like to participate in this study?	
**First, we would like to know more about you.**		
2.1	Ask participant if you can take photo of him/her	
2.2	What is your sex?	1) Male<br>2) Female
2.3	What is your age? (in years)	
2.4	What is your ethnicity?	1) Khmer<br>2) Cham<br>3) Vietnamese<br>4) Chinese<br>5) Other, please specify
2.5	What is your current marital status?	1) Never married (Single)<br>2) Married<br>3) Separated or divorced<br>4) Widowed
2.6	How are you related to the head of household?	1) Myself<br>2) Spouse<br>3) Partner<br>4) Son/Daughter<br>5) Son-in-law/Daughter-in-law<br>6) Parent<br>7) Sibling (e.g. brother, sister, stepbrother, stepsister)<br>8) Grandparent<br>9) Grandchild<br>10) Other relatives (e.g. aunt, uncle, cousin, niece, nephew etc.)<br>11) Hired help<br>12) Friend<br>Others, please specify
2.7	Participant ID	Scan QR code
**Now we'll ask you about some information about your household**		
3.1	How many members live in this household on a typical day?	
3.2	Does this household own any livestock, herds, other farm animals, or poultry?	1) Yes<br>2) No (go to end of survey)

3.3	Who is the main person in this household who makes decisions about animal healthcare?	1) Myself<br>2) Husband<br>3) Wife<br>4) Children<br>5) Siblings<br>6) Parent<br>7) Hired help<br>8) Neighbour<br>9) Other villagers<br>10) Others, please specify
**Now we'll ask you some questions about your animal management:**		
4.1	Do you own any of the following animals?	1) Water buffaloes<br>2) Cows/bulls<br>3) Goats/sheep<br>4) Pigs<br>5) Chickens<br>6) Ducks<br>7) Dogs/cats<br>8) Other, please specify
4.2	Do you take care of any of the following animals outside of your household?	9) Water buffaloes<br>10) Cows/bulls<br>11) Goats/sheep<br>12) Pigs<br>13) Chickens<br>14) Ducks<br>15) Dogs/cats<br>Other, please specify
4.3	How many of ___ do you own?	
4.4	On a typical day, who is the main person who takes care of ___?	1) Myself<br>2) Husband<br>3) Wife<br>4) Children<br>5) Siblings<br>6) Parent<br>7) Hired help<br>8) Neighbour<br>9) Other villagers<br>10) Others, please specify
4.5	On a typical day, how much time is spent feeding ___ (in minutes)?	
4.6	On a typical day, how much time is spent cleaning ___ (in minutes)?	
4.7	On a typical day, how much time is spent taking ___ from home to grazing areas and back (in minutes)?	
4.8	Can you rank your three main sources of income?	1) Livestock sales<br>2) Crop, vegetable, fruit sales<br>3) Animal products' sales<br>4) Self-employment<br>5) Salaries, wages of household members<br>6) Social welfare grants<br>7) Housing and land rent

		8) Other, please specify
4.9	Time end	
