# Supplementary material for: Understanding networks in rural Cambodian farming communities and how they influence antibiotic use: A mixed methods study
Source: PLOS Glob Public Health. 2023 Mar 8;3(3):e0001569. doi: 10.1371/journal.pgph.0001569 (PMC10021636; doi:10.1371/journal.pgph.0001569)
Supplement: S2 Appendix — (PDF) [file pgph.0001569.s003.pdf]

S2 APPENDIX: NETWORK SURVEY QUESTIONNAIRE

**Start network questionnaire**		
1.1	Name of data collector	
1.2	Time start	
1.3	Date	
1.4	Household ID	Scan QR code
1.5	Participant ID	Scan QR code
**Antibiotic knowledge questions**		
2.1	Have you heard about the term "antibiotics"?	1) Yes<br>2) No
	Have you heard of the following medicines?	
2.2	Amoxicillin	1) Yes<br>2) No
2.3	Penicillin	
2.4	Tetracycline	
2.5	Augmentin	
2.6	Ampicillin	
**Antibiotic attitudes and practices (Human)**		
3.1	Have you kept supply of antibiotics at home whether you're sick or not? (prompt: for participants who haven't heard about the term antibiotics, explain that the options above are all types of antibiotics)	1) Yes<br>2) No
3.2	Do you have antibiotics at home right now?	If yes, take photograph of participants' antibiotics
3.3	Have you shared antibioitcs with your family and friends when they are sick with the same symptoms?	1) Yes<br>2) No
3.4	Have you shared antibiotics with your family and friends when they are sick with different symptoms?	1) Yes<br>2) No
3.5	Have you kept leftover antibiotics and used them again when you fell sick?	1) Yes<br>2) No
3.6	Do you usually get a prescription before you buy antibiotics for yourself or for your family?	1) Yes<br>2) No
3.7	Do you use antibiotics for the following? (you can tick more than one)	1) Yes<br>2) No
**Antibiotic attitudes and practices (Animal)**		
4.1	Have you shared your antibiotics with your animals when they are sick?	1) Yes<br>2) No
4.2	Have you fed your animals antibiotics to prevent them from getting sick?	1) Yes<br>2) No
4.3	Have you fed your animals antibiotics to help them grow faster?	1) Yes<br>2) No

4.4	Do you usually get a prescription before you give your animals antibiotics?	1) Yes<br>2) No
4.5	Do you use antibiotics in your animals when they are sick?	1) Yes<br>2) No
4.6	Do you use antibiotics in your animals when they are injured?	1) Yes<br>2) No
4.7	For which animal diseases do you use antibiotics?	1) Food and mouth disease<br>2) Newcastle disease<br>3) Cholera<br>4) Respiratory illness<br>5) Fever<br>6) Pain<br>7) None<br>8) Other, please specify
4.8	What do you currently do to prevent your animals from getting sick? (select all that apply)	1) I keep them clean<br>2) I keep their surroundings clean<br>3) High quality feed<br>4) Feeding antibiotics (not for illness)<br>5) Clean water<br>6) Traditional remedies<br>7) Vaccinations<br>8) Biosecurity practice<br>9) Confinement practice<br>10) Other, please specify
**Vaccination (for animals)**		
5.1	I vaccinate my:	1) Cows/bulls<br>2) Horses/donkeys/mules<br>3) Goats/sheep<br>4) Pigs<br>5) Chickens<br>6) Ducks
5.2	Which conditions do you vaccinate your ___ for?	1) Food and mouth disease<br>2) Newcastle disease<br>3) Cholera<br>4) Classical Swine Fever (CSF)<br>5) Pasteurellosis<br>6) Salmonellosis<br>7) PRRS<br>8) Black leg<br>9) Other, please specify
**Antimicrobial Resistance (AMR) knowledge**		
6.1	Have you heard of any of the following terms? (you can tick more than one)	1) Antibiotic resistance<br>2) Antimicrobial resistance<br>3) Drug resistance<br>4) AMR<br>5) Antibiotic-resistant bacteria<br>6) Never heard of any before (Skip section)
6.2	Where have you heard the terms from? (you can tick more than one)	1) Doctor<br>2) Nurse<br>3) Midwife<br>4) Pharmacist

		5) Pett<br>6) Public hospital<br>7) Health centre<br>8) Family members<br>9) Friends<br>10) Media<br>11) Specific campaigns<br>12) Other, please specify<br>13) Can't remember
6.3	Antibiotic resistance occurs when your body becomes resistant to antibiotics and they no longer work as well	1) Yes<br>2) No<br>3) Unsure
6.4	Many infections are becoming increasingly resistant to treatment by antibiotics	1) Yes<br>2) No<br>3) Unsure
6.5	If bacteria are resistant to antibiotics, it can be very difficult or impossible to treat the infections they cause	1) Yes<br>2) No<br>3) Unsure
6.6	Antibiotic resistance is an issue that could affect me or my family	1) Yes<br>2) No<br>3) Unsure
6.7	Antibiotic resistance is an issue in other countries but not here	1) Yes<br>2) No<br>3) Unsure
6.8	Antibiotic resistance is only a problem for people who take antibiotics regularly	1) Yes<br>2) No<br>3) Unsure
6.9	Bacteria which are resistant to antibiotics can be spread from person to person	1) Yes<br>2) No<br>3) Unsure
6.10	Antibiotic-resistant infections could make medical procedures like surgery, organ transplants and cancer treatment much more dangerous.	1) Yes<br>2) No<br>3) Unsure
6.11	Antibiotic resistance can spread between animals and humans	1) Yes<br>2) No<br>3) Unsure
6.12	What are the consequences of antibiotic resistance? (You can tick more than one option)	1) Difficult to treat infections<br>2) More diseases appearing<br>3) More money and time is spent<br>4) More patients might die<br>5) Not sure
**Network relationships**		
7.1	Can you name up to 3 people who you spend the most time talking to on a typical day?	Scan QR codes
**Work related matters**		
7.2	Can you name up to 3 people who you trust to discuss important work-related matters with?	Scan QR codes

7.3	Can you name up to 3 people who you work with most closely on the farm?	Scan QR codes
7.4	Can you name up to 3 people who you trust to manage your farm when you are unable to?	Scan QR codes
7.5	Do you buy commercial feed for your animals?	1) Yes<br>2) No
7.6	Who do you usually buy commercial animal feed from?	1) Family and friends<br>2) Grocery store<br>3) Convenience store<br>4) Animal health worker<br>5) Animal feed store<br>6) NGO<br>7) Other, please specify
7.7	Can you name up to 3 people who you buy commercial animal feed from?	Scan QR codes (if family and friends were chosen)
7.8	Do you sell your animals? (either for food/for other farmers to raise etc.)	1) Yes<br>2) No
7.9	Can you name up to 3 people who help you to sell your animals?	Scan QR codes
**Health**		
7.10	Can you name up to 3 people who you ask for advice about general health-related matters?	Scan QR codes
7.11	Thinking about the last time you got antibiotics, where did you get them from?	1) Family and friends<br>2) Grocery store<br>3) Gas station<br>4) Convenience store<br>5) Doctor<br>6) Pett<br>7) Pharmacy<br>8) Private clinic<br>9) Health centre<br>10) Hospitals<br>11) Other, please specify
7.12	Can you name up to 3 family members/friends that you got your antibiotics from?	Scan QR codes
7.13	How did you decide on the type of antibiotics to get? (you can tick more than one)	1) Prescription from doctors<br>2) I decided on my own<br>3) Advice from the person you purchased from<br>4) Advice from the Pett<br>5) Advice from lab staff<br>6) Advice from friends<br>7) Advice from family<br>8) Media<br>9) Other, please specify
7.14	Can you name up to 3 family members/friends that you got your advice from?	Scan QR codes (if family and friends were chosen)
**Animal health**		

7.15	Can you name up to 3 people who you ask for general advice about raising your animals?	Scan QR codes
7.16	Can you name up to 3 people who you ask for advice about preventing your animals from getting sick?	Scan QR codes
7.17	Can you name up to 3 people who you ask for advice when you need help dealing with your sick animals?	Scan QR codes
7.18	Can you name up to 3 people who you ask for advice when you need help dealing with dead animals?	Scan QR codes
7.19	Thinking about the last time you got antibiotics for your animals, where did you get them from?	1) Family and friends<br>2) Grocery store<br>3) Gas station<br>4) Convenience store<br>5) Doctor<br>6) Pett<br>7) Pharmacy<br>8) Private clinic<br>9) Health centre<br>10) Hospitals<br>11) Other, please specify
7.20	Can you name up to 3 family members/friends that you got your antibiotics from?	Scan QR codes (If family and friends were chosen)
7.21	How did you decide on the type of antibiotics to get? (you can tick more than one)	1) Advice from the animal health worker<br>2) I decided on my own<br>3) Advice from the person you purchased it from<br>4) Advice from the Pett<br>5) Advice from friends<br>6) Advice from family<br>7) Media<br>8) NGO<br>9) Other, please specify
7.22	Can you name up to 3 family members/friends that you got your advice from?	Scan QR codes (if family and friends were chosen)
7.23	If you need vaccines for your animals, who/where do you get them from?	1) I do not vaccinate my animals<br>2) Family and friends<br>3) Grocery store<br>4) Convenience store<br>5) Pett<br>6) Pharmacy<br>7) Private clinic<br>8) Health centre<br>9) Hospitals<br>10) Animal health worker<br>11) Animal feed store<br>12) NGO<br>13) Other, please specify

7.24	Can you name up to 3 family members/friends that you get your vaccines from?	Scan QR codes (if family and friends were chosen)
7.25	Time end	
