# Supplementary material for: Understanding networks in rural Cambodian farming communities and how they influence antibiotic use: A mixed methods study
Source: PLOS Glob Public Health. 2023 Mar 8;3(3):e0001569. doi: 10.1371/journal.pgph.0001569 (PMC10021636; doi:10.1371/journal.pgph.0001569)
Supplement: S3 Appendix — (PDF) [file pgph.0001569.s004.pdf]

S3 APPENDIX: IN-DEPTH INTERVIEW TOPIC GUIDE

Before we start the interview formally, we'd like to …

Participant background

1) How long have you lived in this village? (been a village animal health worker in this village)
    a. [if they have animals] What kind of animals do you have/do you treat?
2) Can you describe a typical day for me?
    a. Probing questions can include the people they see in their daily routine etc.

Now we'd like to ask you some questions about caring for your animals,

Participant's knowledge/use of antibiotics

1) What are some common conditions/symptoms that you see in your cattle/chickens/ducks etc?
    a. Over the past six months, how often did your animals get sick? How did this affect your income?
2) When your animals get sick, what do you usually do? (if they have different types of animals, ask for each one) [interviewer: If they mention medications, ask about antibiotics]
    a. What symptoms or signs in your animals prompt you to use antibiotics?
    b. Before you use antibiotics, do you try anything else? (e.g. prompt improve hygiene, probiotics etc.)
    c. How do you feed antibiotics to your animals in these instances? (probing questions about frequency/dosage)
3) Besides using antibiotics for illness, what else comes to mind when you hear 'antibiotics'?
4) Do you use them for preventing illness/growing faster etc?
    a. If yes, where did you learn that from?
    b. How do you feed antibiotics to your animals in these instances? (probing questions about frequency/dosage)
    c. How effective have you found this to be?
5) What kinds of antibiotics do you usually use for your animals, whether to treat or prevent illness in animals? (to find out if participants specifically buy antibiotics for their animals or if they use human antibiotics)
    a. [if they use human antibiotics] Why do you use human antibiotics?
    b. How effective have you found this to be?
6) Besides antibiotics, what do you think influences/prevents illnesses in yourselves or your animals?
    a. Where did you learn/who did you learn these from?
    b. How effective have you found these methods to be?
    c. Have you used antibiotics to prevent yourself from getting sick?
7) Where did you initially learn to care for your animals? How did you learn to care for animals?
    a. Probing questions can include dietary advice, sickness, getting rid of dead animals etc.

Selling antibiotics/animal feed store?

1) What kinds of antibiotics do you usually sell?
2) Where do you get these antibiotics from?
3) How do people generally buy medicines from you?

a. E.g. do people usually tell you what antibiotics/medicines they want or do they tell you symptoms…

Now we're going to use this mat to explore some of the relationships that you have with others in this village…

Participant's relationships

1) Previously in the survey we asked you about who **you generally spend the most time talking to** vs. **talking about something personal/private**…
    a. (if they're different) how do you decide?
    b. With the people who you talk to about something personal/private, can you rank them in terms of who you talk to/see most frequently?
    c. Can you rank them in terms of who you trust to keep this information private?
    d. Can you now rank them in terms of who gives good advice?
    e. *(interviewer: if there are any changes, please explore those changes with participants)*

2) Can you name up to 3 people who you talk to about **important work-related matters** vs. Can you name up to 3 people who you **work with most closely on the farm?**
    a. (if they're different) how do you decide?
    b. With the people you talk to about important work-related matters, can you rank them in terms of who you talk to/see most frequently?
    c. Can you rank them in terms of who generally gives good advice about your work-related matters?
    d. *(interviewer: if there are any changes, please explore those changes with participants)*

3) Can you name up to 3 people who you **work with most closely on the farm?** vs. Can you name up to 3 people who you trust to **manage your farm when you are unable to?**
    a. (if they're different) how do you decide? What are some factors that you take into consideration when you let someone else manage your farm?

4) Can you name up to 3 people who you ask for advice about **general health-related matters?**
    a. (if the participant has previously named multiple people) Can you rank them in terms of how often you see them?
    b. Can you now rank them in terms of the quality of advice that they give?
    c. Can you now rank them in terms of how much you trust the advice that they give?
    d. *(interviewer: if there are any changes, please explore those changes with participants)*

5) (if they are influential in this area) Who do you generally give advice about **health-related matters to?**
    a. What are some common things that people ask you advice about?
    b. How do you usually respond (for certain conditions)?
    c. Where did you learn about …

6) Can you name up to 3 people who you ask for advice about **general health-related matters?** vs. Can you name up to 3 people who you ask for **general advice about raising your animals?**
    a. (If they're different) how do you decide?

b. With the people you ask for general advice about raising your animals, can you rank them in terms of how often you see them?
c. Can you now rank them in terms of the quality of advice that they give?
d. Can you now rank them in terms of how much you trust the advice that they give?
e. *(interviewer: if there are any changes, please explore those changes with participants)*

7) (if they are influential in this area) Who do you generally give advice about **raising animals?** (To ask village animal health worker too)
    a. What are some common things that people ask you advice about?
    b. How do you usually respond (for certain conditions)?
    c. Where did you learn about …

8) Can you name up to 3 people who you ask for **general advice about raising your animals?** vs. Thinking about the last time you got antibiotics for your animals…how did you decide what type of antibiotics to get?
    a. (If they're different) how do you decide?
    b. With the people you ask for general advice about raising your animals, can you rank them in terms of how often you see them?
    c. Can you now rank them in terms of the quality of advice that they give?
    d. Can you now rank them in terms of how much you trust the advice that they give?
    e. *(interviewer: if there are any changes, please explore those changes with participants)*
