# Supplementary material for: Understanding networks in rural Cambodian farming communities and how they influence antibiotic use: A mixed methods study
Source: PLOS Glob Public Health. 2023 Mar 8;3(3):e0001569. doi: 10.1371/journal.pgph.0001569 (PMC10021636; doi:10.1371/journal.pgph.0001569)
Supplement: Attachment — Submitted filename: reviewercomments_01.docx [file pgph.0001569.s006.docx]

Journal Requirements:

1. Please include a complete copy of PLOS’ questionnaire on inclusivity in global research in your revised manuscript. Our policy for research in this area aims to improve transparency in the reporting of research performed outside of researchers’ own country or community. The policy applies to researchers who have travelled to a different country to conduct research, research with Indigenous populations or their lands, and research on cultural artefacts. The questionnaire can also be requested at the journal’s discretion for any other submissions, even if these conditions are not met.  Please find more information on the policy and a link to download a blank copy of the questionnaire here: https://journals.plos.org/globalpublichealth/s/best-practices-in-research-reporting. Please upload a completed version of your questionnaire as Supporting Information when you resubmit your manuscript.

We have uploaded a complete version of our questionnaire as Supporting Information.

2. Please amend your detailed Financial Disclosure statement. This is published with the article. It must therefore be completed in full sentences and contain the exact wording you wish to be published.

a. State the initials, alongside each funding source, of each author to receive each grant.

b. State what role the funders took in the study. If the funders had no role in your study, please state: “The funders had no role in study design, data collection and analysis, decision to publish, or preparation of the manuscript.”

c. If any authors received a salary from any of your funders, please state which authors and which funders.

If you did not receive any funding for this study, please simply state: “The authors received no specific funding for this work.”"

Thanks for the comment. We have included the details of the funding source and the role the funders took in the study in our financial disclosure statement (highlighted on page 13).

3. Please provide separate figure files in .tif or .eps format only and remove any figures embedded in your manuscript file. Please also ensure that all files are under our size limit of 10MB.

For more information about figure files please see our guidelines:

https://journals.plos.org/globalpublichealth/s/figures

https://journals.plos.org/globalpublichealth/s/figures#loc-file-requirements

We have provided separate figure files in .tiff in our resubmission.

4. We have noticed that you have uploaded Supporting Information files, but you have not included a list of legends. Please add a full list of legends for your Supporting Information files after the references list.

We have added a list of legends for the supporting information. This is highlighted on page 17.

5. Figures 2 & 4: Please confirm (a) that you are the photographer; or (b) provide written permission from the photographer to publish the photo(s) under our CC-BY 4.0 license.

Thanks for the clarification. We confirm that last author Clarence Tam is the photographer for both figures 2 and 4.

6. Figure 1: please (a) provide a direct link to the base layer of the map (i.e., the country or region border shape) and ensure this is also included in the figure legend; and (b) provide a link to the terms of use / license information for the base layer image or shapefile. We cannot publish proprietary or copyrighted maps (e.g. Google Maps, Mapquest) and the terms of use for your map base layer must be compatible with our CC-BY 4.0 license.

Note: if you created the map in a software program like R or ArcGIS, please locate and indicate the source of the basemap shapefile onto which data has been plotted.

If your map was obtained from a copyrighted source please amend the figure so that the base map used is from an openly available source. Alternatively, please provide explicit written permission from the copyright holder granting you the right to publish the material under our CC-BY 4.0 license.

Please note that the following CC BY licenses are compatible with PLOS license: CC BY 4.0, CC BY 2.0 and CC BY 3.0, meanwhile such licenses as CC BY-ND 3.0 and others are not compatible due to additional restrictions.

If you are unsure whether you can use a map or not, please do reach out and we will be able to help you. The following websites are good examples of where you can source open access or public domain maps:

* U.S. Geological Survey (USGS) - All maps are in the public domain. (http://www.usgs.gov)

* PlaniGlobe - All maps are published under a Creative Commons license so please cite “PlaniGlobe, http://www.planiglobe.com, CC BY 2.0” in the image credit after the caption. (http://www.planiglobe.com/?lang=enl)

* Natural Earth - All maps are public domain. (<http://www.naturalearthdata.com/about/terms-of-use/>)

The map from Figure 1 was created in R using the package ­*rnaturalearth* (South, 2017). This package uses maps from Natural Earth where all maps are public domain.

Additional Editor Comments (if provided):

Your manuscript has now been assessed by two independent reviewers and while both reviewers see merit in the work, both raise concerns that would preclude publication in the current form. Both reviewers appreciated the mixed method approach used in the study, but I agree with reviewer #2 that many of the findings are overstated and not supported by the data collected and sections of the discussion will need to be revised in an updated version of the manuscript. Both reviewers also noted low numbers for some statistical tests that need to be caveated.

Reviewers' comments:

5. Review Comments to the Author

Please use the space provided to explain your answers to the questions above. You may also include additional comments for the author, including concerns about dual publication, research ethics, or publication ethics. (Please upload your review as an attachment if it exceeds 20,000 characters)

Reviewer #1: This interesting mixed-methods study explores factors influencing antibiotic use in humans and animals in rural Cambodia.

Strengths of the manuscript include:
- It is well written
- The methodology is sophisticated and well described
- The findings are an important contribution to the literature

Title:
- I would suggest adding the study type (mixed methods)

Methods:
- Some information on how the villages were selected would be interesting
- USD3 => USD 3 or 3 USD
- “No members of the public were involved in the study.” In the study design ?

Results:
- “and most commonly owned buffaloes and chickens” probably cows instead of buffaloes (only one household seemed to have owned a buffalo based on the table 1)
- In addition to percentages, it would be good to also always add numbers “n”
- Is any information available on how often participants misidentified other medicines as antibiotics

Thank you for the constructive comments. We have made the following changes:

- Adding study type to the title
- Villages were selected with input from our local collaborators at CelAgrid, namely, these were villages that were previously involved in their agricultural programmes. We have added this information to the Methods section on pg 3 (highlighted in yellow)
- We have changed USD3 and USD8 to USD 3 and USD 8 respectively
- We have also changed the sentence to “No members of the public were involved in the conceptualisation of the study” (highlighted on pg 3) and changed buffaloes to cows (highlighted on pg 5)
- We have added numbers “n” to the results section on pgs 7-8.
- Unfortunately we did not specifically collect information on how often participants misidentified other medicines as antibiotics, but according to photographs collected, about 14 households said that they kept a supply of antibiotics at home whether they were sick or not, but did not have any antibiotics in their medicine supply at home (pg 8).

Reviewer #2: This is an interesting article that addresses the previously documented phenomenon of antibiotic use among Cambodian 'backyard' farmers. It uses a range of methodological approaches including household census, social network survey and in-depth interviews to document local knowledge about antibiotics and social informational ties between villagers. While the results are useful, it is unclear whether the authors really demonstrate 'how [networks] influence antibiotic use' as claimed in the title. A key question is the claim regarding the 'spread of antibiotic behaviours through social contacts and influence' as this survey does not constitute causal evidence. In addition, all data on antibiotic use is self-reported, and responses may well have been affected by the established close links between the livestock centre that facilitated the study and by the somewhat intrusive research approach involving taking photographs of individuals and sharing these as a means to measure social networks.

I am not qualified to comment on the statistical methods used to analyse the social network data which are highlighted in the paper, but it is unclear whey key informants were selected from participants' 'talking' networks rather than their 'health' networks, since the latter was the focus of the study. Although great emphasis is placed in the paper on the social network survey, it is not clear that this adds much to our knowledge beyond verifying the rather well known fact that in small rural communities, kinship and neighbourhood ties are of primary importance for information sharing and social relations.

Thanks for the comment. We used talking networks to inform our key informant selection because it provided the most comprehensive overview of the social networks within both villages (i.e. who were “influential” participants who could reach a significant portion of the network). We then asked these key informants about where they obtained their health information and explored other trusted sources of health information. Through this process, we found out that while kinship, neighbourhood and familial ties are important for information sharing and social relations, there were discrepancies in their nominations for the types of information shared – participants nominated different members of the community for different pieces of information. For instance, expertise and experience were valued more when choosing to turn to another member in the community for health advice and information. Additionally, we also interviewed staff at the health centres in both villages to understand the types of common medical conditions that participants sought advice for.

In the discussion of limitations, it is acknowledged that all information on antibiotic practices is self-reported and this needs to be reflected accurately in the Results section; e.g. instead of asserting that 'participants often relied on their own knowledge...' or 'they used alternative methods...' (p.8) it should be explicitly stated that the participants reported relying on... reported using... etc.

Thanks for the constructive feedback. We have made edits to the Results section to indicate that all participant information on antibiotic practices is self-reported. This can be found on pages 6-8.

Likewise, where results are based on prompts such as the use of biosecurity practices, this should be explicitly noted in the results section, as this otherwise gives the impression that informants volunteered this information autonomously.

We have clarified the statement to reflect that this information was shared during in-depth interviews.

The limitations section should also consider the potential effects of requiring informants to be photographed before participating in the research and of sharing photographs with other villagers.

Thanks for the comment. We have added this limitation to the discussion section (highlighted on page 12).

In the Methods or Antibiotic scores section more details should be provided on how/why statements were selected for inclusion and the basis for developing the scoring system. The Results section is rather short and a number of findings not reported in this section are instead included in the Discussion, such as factors that influenced participants' decisions to obtain antibiotics, and ideas about anitibotic awareness (p.11). These results should be moved to the Results section and then discussed here.

Thanks for the clarification. The scored variables were questions adopted from the WHO multi-country public awareness survey on antibiotic resistance to understand different dimensions of antibiotic behaviours. The scoring is also elaborated on in Appendix 2 as supporting information to the manuscript. We also used these variables for scoring in a previous population survey in Cambodia (<https://www.ncbi.nlm.nih.gov/pmc/articles/PMC8210153/>) and potentially could be used for comparison at different time points. Additionally, we have moved relevant sections to the Results section where they are more pertinent (highlighted).

In addition, some points included in the discussion are not based on any findings reported in the results section, such as the benefits of improved sanitation and waste management on AMR, so the authors sometimes make claims beyond the evidence presented.

Thanks for the comment. We have removed the point in the discussion to ensure that the discussion section is coherent with our findings.
